# Book Review

**Published:** 1914-12

**Authors:** 


**Tabular Diagnosis: An Aid to the Rapid Differential Diagnosis of Diseases.** By RALPH WINNINGTON LEFTWICH, M.D. Pp. vi, 359. London: Edward Arnold. 1913. Price, 7s. 6d. net.—There is a singular difficulty in deciding how far a method of memorisation is in itself good or bad. Some memories, no doubt, are best served by bare lists of words which are apparently photographed into the mind. This work provides an example of such a method carried to a great degree of perfection. A collection of 350 diseases or symptom-complexes reduced each to some ten or twelve salient points is put together, so that pairs of diseases may be rapidly contrasted according to the table of their symptoms. This method fails in the actualities of life, and for this reason, that, for example, "cerebellar tumour" is contrasted only with "ataxic paraplegia," with "hereditary cerebellar ataxia," and with "cerebral tumour"; while another and more difficult task may be to distinguish the condition from mere "labyrinthine" disease. We have found that practitioners who rely upon this class of book are apt to make artificial difficulties based upon the tables they have learnt, to overlook any unforeseen similarity of diseases, and to remain wholly unobservant of unusual symptoms not included in their stereotyped lists and charts. They tend to trust to dead reckoning, and forget to "heave the lead" when in a fog. But the book is well arranged and well printed.

**Medical Electricity.** By H. LEWIS JONES, M.A., M.D. Sixth Edition. Pp. xv, 551. London: H. K. Lewis. 1913. Price, 12s. 6d. net.—Dr. Lewis Jones's *Medical Electricity* is by now the established hand-book in English on this subject. This the latest edition serves to emphasise the fact. The author must feel some satisfaction at the firmer foundation upon which electro-therapeutics now stands. To this he has himself largely contributed by his happy combination of scientific knowledge, clinical acumen and literary elegance.

**The Clinical Pathology of Syphilis and Parasyphilis, and its Value for Diagnosis and Controlling Treatment.** By HUGH WANSEY BAYLY, M.A., M.R.C.S., L.R.C.P. Pp. xiii, 194. London: Baillière, Tindall & Cox. 1912. Price, 5s. net.—The author, who is pathologist to the London Lock Hospital, has written an excellent little manual which will prove of the greatest assistance to the clinician. The modern developments in the diagnosis of syphilis are not sufficiently widely known or understood, nor is their importance adequately insisted upon by those responsible for the instruction and examination of students. We recommend this book as being a short and intelligible explanation of the technique of simple clinical pathology and its bearing upon the diagnosis and treatment of syphilis.

**Our Outsides, and what they Betoken.** A Summary by W. T. Fernie, M.D. Pp. xi, 407. Bristol: John Wright and Sons Ltd. 1913. Price, 4s. 6d.—Dr. Fernie bids fair to rival the immortal anatomist of melancholy in his merry and facete writings. Hitherto it has been possible in a review to convey some hint of the contents of one of his books. Now, however, we confess to defeat. Temperaments, physiognomy, phrenology, manners and fashions, clothes and complexions, form the burden of this volume. But to analyse and explain how it comes to form such pleasant reading would be like inquring what goes into *Bouilla baisse.* In Anthony Wood's words, " 'Tis a book so full of variety of reading, that gentlemen who have lost their time and are put to a push for invention, may furnish themselves with matter for common or scholastical discourse and writing."

**Diseases of the Heart.** By James Mackenzie, M.D., F.R.C.P. Pp. xxiii, 502. London: Oxford Medical Publications. 1913. Price, 25s. net.—The main changes in the third edition of this work lie in the illustrations derived from electro-cardiograms. The correlation of *post-mortem* investigations with clinical observations made under modern conditions has enabled the author to throw additional light upon obscure cardiac symptoms and syndromes. Each new edition of Mackenzie on the Heart is beginning to be looked for with eager interest.

**Lectures on Medical Electricity to Nurses.** By J. Delpratt Harris, M.D. Durh., M.R.C.S. Pp. ix, 88. London: H. K. Lewis. 1913. Price, 2s. 6d. net.—For an elementary course of instruction illustrated by practical demonstration this book is well planned. It is doubtful whether the mere reading of it by any nurse would add anything to her knowledge of the subject, unless she had attended some practical classes. Certainly, after the rudiments of electricity have been mastered, much assistance could be obtained from these lectures. The practical hints called " useful wrinkles " at the end of the book are sound and well expressed. The price is moderate, and the printing and illustrations are good

**Natural Therapy: a Manual of Physiotherapeutics and Climatology,** By Thomas D. Luke, M.D., F.R.C.S. Edin., and Norman Hay Forbes, F.R.C.S. Edin., and F.R.S. Edin.. Pp. xvi, 316. Bristol: John Wright & Sons Ltd. 1913. Price 5s. net.—We are glad to see a second edition of this interesting introduction to physical therapeutics. The claims of hydrotherapy, thermotherapy, and other natural agents are put forward with reason and moderation, and the criticism of opponents is not forgotten. We may give as instances of this fairness the accounts of the Nauheim Baths and of High Frequency Currents. The relation of cutaneous areas to internal organs by vascular as well as nerve reflexes appears rather peculiar. But the authors give a vivid and useful account of the methods used in the large hydropathic and thermal establishments, methods which have been found successful, and of which most of us are profoundly ignorant. A new feature in this edition is a chapter on climatology from the pen of Dr. Forbes, packed with common-sense suggestions as to the choice of climates and watering-places for invalids. In a small volume such as this omissions must be expected, but a valuable bibliography of each subject shows where detailed information should be sought for. One would have thought, however, that space would have been found for a fuller account of the cold bath treatment in fevers, where hydrotherapists have scored their greatest triumph. A section on ionization might well have replaced much of the space taken up by descriptions of electric batteries, and we should like a clearer statement of the necessity when working with currents from the main of always employing a secondary current, or one from a motor transformer, even for local baths. It is so easy even in a private house to pass a sinusoidal lighting current through a sledge coil and lamp, and thus get a safe and pleasant electric bath. One of the best and most interesting sections in the book is the discussion on the relative values of luminous and non-luminous heat and their physiological effects. In the chapters on diet it is curious to find cropping up the unfounded preference for toast over bread in mild cases of diabetes, and the absence of the Lenhartz diet in gastric ulcer. The authors have certainly succeeded in giving a large amount of information in a manner calculated to awaken much interest in their subject.

**The Fæces of Children and Adults.** By P. J. Cammidge, M.D. Pp. viii, 516. Bristol: John Wright & Sons Ltd. 1914. 17s. 6d. net.—This book contains an account of the methods of microscopical, bacteriological and chemical examinations of the fæces, together with a consideration of the diagnostic value of such work, and some indications for treatment. The index is very thorough, and there is a useful appendix giving various forms of dietaries. The author states that the book is chiefly a compilation from Hecht's "*Die Fæces des Sauglings und des Kindes,*" and Schmidt and Strasburger's "*Fæces des Menschen.*" Dr. Cammidge has, however, added some observations of his own. Each chapter has its own bibliography. The volume will be found useful for reference, but it has not been written in a manner particularly helpful to the laboratory worker. Perhaps this will be remedied in a future edition. The illustrations are a little crude in colour and design, and it is a question whether they will be of much value to the student or practitioner. Although we are not quite in accord with the form in which this book is cast, we recognise the evident care in preparation, and the value of the record of details. There will be many a tribute of thanks for the unsparing energy and thought which has resulted in the publication of an English book of reference on this subject. Very probably, however, the author will himself admit that the whole subject is being extended unnecessarily, and that he has been compelled to submit a compilation of a large number of comparatively isolated facts, difficult to digest, and almost beyond the powers of memory. Some chapters containing a critical survey of methods and observations, and a short résumé of the more general principles concerned might have been added with advantage.

**Thomas Shortt.** By Arnold Chaplin, M.D. Pp. 70. London: Stanley Paul & Co. 1914. Price 2s.—The author of *The Illness and Death of Napoleon*, now gives us a history of the principal medical officer in St. Helena, with biographies of some other medical men associated with the case of Napoleon from 1815 to 1821. We have no wish to re-open the unseemly controversy surrounding the illness and death of the first Napoleon, but the fact that Dr. Shortt was in 1820 appointed as Principal Medical Officer to the King's Forces in St. Helena brought him into the controversy, and formed the most notable event in his military career. He was in charge of the proceedings at the *post-mortem* examination, and the following statement made by him shows how immaterial was the controverted question as to some enlargement of the liver. He wrote: "In opening the body it presented an unusual quantity of fat, and every part of it was sound, excepting the stomach, which was a perfect mass of disease from cancer, and ulcerated in several places. In one place near the lower opening there was a hole sufficiently large to admit the little finger, which penetrated the coats of the stomach, and which was attached to the liver so that the contents were prevented from escaping." Shortt appears to have been unjustly criticised, for his only crime was that he had the courage to state what he honestly believed, and not what the official minds desired. After leaving St. Helena in 1821 Dr. Shortt resumed his practice in Edinburgh, where he became a physician of considerable note. In 1842 he became Inspector of Prisons in Great Britain, but was attacked with pneumonia in 1843, and died in the fifty-fifth year of his age.

